# Feasibility of using alternative swabs and storage solutions for paired SARS-CoV-2 detection and microbiome analysis in the hospital environment

**DOI:** 10.21203/rs.3.rs-56028/v2

**Published:** 2020-12-15

**Authors:** Jeremiah Minich, Farhana Ali, Clarisse Marotz, Pedro Belda-Ferre, Leslie Chiang, Justin P. Shaffer, Carolina S. Carpenter, Daniel McDonald, Jack Gilbert, Sarah M. Allard, Eric E Allen, Rob Knight, Daniel A. Sweeney, Austin D. Swafford

**Affiliations:** University of California San Diego Scripps Institution of Oceanography; University of California San Diego; University of California San Diego; University of California San Diego; University of California San Diego; University of California San Diego; University of California San Diego; University of California San Diego; University of California San Diego; University of California San Diego; University of California San Diego Scripps Institution of Oceanography; University of California San Diego; University of California San Diego

**Keywords:** COVID-19, SARS-CoV-2, RT-qPCR, swab, global health

## Abstract

**Background:**

Determining the role of fomites in the transmission of SARS-CoV-2 is essential in the hospital setting and will likely be important outside of medical facilities as governments around the world make plans to ease COVID-19 public health restrictions and attempt to safely reopen economies. Expanding COVID-19 testing to include environmental surfaces would ideally be performed with inexpensive swabs that could be transported safely without concern of being a source of new infections. However, CDC-approved clinical-grade sampling supplies and techniques using a synthetic swab are expensive, potentially expose laboratory workers to viable virus and prohibit analysis of the microbiome due to the presence of antibiotics in viral transport media (VTM). To this end, we performed a series of experiments comparing the diagnostic yield using five consumer-grade swabs (including plastic and wood shafts and various head materials including cotton, synthetic, and foam) and one clinical grade swab for inhibition to RNA. For three of these swabs, we evaluated performance to detect SARS-CoV-2 in twenty intensive care unit (ICU) hospital rooms of patients including COVID-19+ patients. All swabs were placed in 95% ethanol and further evaluated in terms of RNase activity. SARS-CoV-2 was measured both directly from the swab and from the swab eluent.

**Results:**

Compared to samples collected in VTM, 95% ethanol demonstrated significant inhibition properties against RNases. When extracting directly from the swab head as opposed to the eluent, RNA recovery was approximately 2-4x higher from all six swab types tested as compared to the clinical standard of testing the eluent from a CDC-approved synthetic (SYN) swab. The limit of detection (LoD) of SARSSARS-CoV-2 from floor samples collected using the consumer-grade plastic (CGp) or research-grade plastic The Microsetta Initiative (TMI) swabs was similar or better than the SYN swab, further suggesting that swab type does not impact RNA recovery as measured by the abundance of SARSSARS-CoV-2. The LoD for TMI was between 0-362.5 viral particles while SYN and CGp were both between 725-1450 particles. Lastly microbiome analyses (16S rRNA gene sequencing) of paired samples (nasal and floor from same patient-room) collected using different swab types in triplicate indicated that microbial communities were not impacted by swab type, but instead driven by the patient and sample type.

**Conclusions:**

Compared to using a clinical-grade synthetic swab, detection of SARS-CoV-2 from environmental samples collected from ICU rooms of patients with COVID was similar using consumer grade swabs, stored in 95% ethanol. The yield was best from the swab head rather than the eluent and the low level of RNase activity and lack of antibiotics in these samples makes it possible to perform concomitant microbiome analyses.

## Background

Since its appearance in early December of 2019, Severe acute respiratory syndrome coronavirus 2 (SARS-CoV-2), the causative agent of coronavirus disease 2019 (COVID-19), has spread to 197 countries resulting in a total of 1,465,144 deaths and 62,844,837 confirmed cases as of December 1, 2020 [1]. As health officials rush to contain the spread of the disease, federal governments are combating the economic fallout, and there is a pressing need to reopen the economies albeit safely, gradually, and in stages. Large-scale testing and contact tracing remain key for controlling viral spread. In addition, environmental sampling of microbes can support the epidemiologic investigations of disease outbreaks [2-4] and shows promise for monitoring SARS-CoV-2 [5]. However, there are supply and cost limitations with the products currently recommended required by the U.S. Centers for Disease Control (CDC) and World Health Organization (WHO) protocols for sample collection supplies [4,6].

The recommendations for human testing of SARS-CoV-2 by the Centers for Disease Control and Prevention (CDC) has varied as the pandemic had unraveled, with updates to methods and sites of sampling, necessary personal protective equipment and supplies, and isolation guidelines. The current guidelines for initial diagnostic testing of human subjects recommends a trained healthcare personnel to collect an upper respiratory specimen, with the swab placed into a sterile tube containing viral transport medium (VTM), Ames transport medium, phosphate buffered saline, or sterile saline [7]. As for surfacing sampling of SARS-CoV-2, the World Health Organization released a sampling protocol recommending environmental samples be taken using a synthetic tipped swab with a plastic shaft collected into a vial with VTM including neutralizing buffer, or chaotropic lysis buffers should transport conditions not be optimal [7]. Given these requirements, and the ongoing surge of cases, clinical-grade synthetic swabs and viral transport medium (VTM)\ are being depleted in developed nations like the United States, and are in even shorter supply in resource limited settings including low- and middle-income countries [4]. Broad SARS-CoV-2 surveillance requires inexpensive, readily available swabs and collection reagents for microbiologic surface fomite sampling protocols requires to support the large sample sizes at geographic scales necessary to inform public health policy. The growing need for environmental testing will place additional demands on current swab supplies.

The use of the CDC-recommended VTM places an additional barrier to efficient and safe deployment of screening and sampling measures. VTM maintains viral viability and therefore the CDC recommends that all samples be handled in a biosafety level-2 (BSL-2) laboratory. VTM also contains antimicrobial agents that limit the type of research studies into likely to interfere with downstream assessment of the microbial context of SARS-CoV-2, such as microbial relationships with that may enable new insights into viral susceptibility and resistance as demonstrated by several recent reports [5,8-10]. Using inactivating sample collection solutions, such as microbiome assay-compatible alcohols, would increase the number of testing laboratories capable of performing SARS-CoV-2 screening, and ameliorate the risks associated with sample transport and processing. Given these considerations, validation of alternative strategies such as self-administered testing using consumer-grade materials and inactivating storage media is urgently needed.

There are aspects of both the swab and the transport media which must be considered when developing a testing procedure for SARS-CoV-2. From a microbiome perspective, the primary concern with using alternative media and consumer-grade materials is the risk of contaminant RNases and/or PCR inhibitors. The presence of these molecules would increase the false negative rate of detecting SARS-CoV-2 RNA by either degrading the virus, or interfering with reverse transcription and quantitative polymerase chain reactions (RT-qPCR) which are the basis for SARS-CoV-2 testing [11]. In addition, the ability to extract the virus from either the swab or the swab eluent must be elucidated as the fixative property of alcohols could result in nucleic acids adhering to swab heads, reducing the ability to measure SARS-CoV-2 RNA from the swab eluent [9]. To fully address these concerns, large screening efforts comparing the recommended and alternative collection methods are needed. However, given the present scale and urgency of the COVID-19 pandemic outbreak, limiting this comparison to a small number of viable options would greatly expedite providing guidance for alternatives to the supply chain this process while minimizing costs. Here we characterize the suitability of detecting SARS-CoV-2 RNA in experimental conditions as well as COVID-19 patient and built-environment samples using viral-inactivating storage solutions and alternative medical-grade and consumer-grade swabs.

## Materials And Methods

### VTM versus EtOH sample comparison

Two cohorts were used to compare the efficacy of SARS-CoV-2 detection in samples collected in VTM compared to EtOH using the same CDC-approved sterile synthetic rayon head, plastic-shaft (‘SYN’, BBL Culture swab REF-220135, Becton, Dickinson and Company) swab. The first cohort of samples was from the Center for Advanced Laboratory Medicine (CALM) at UC San Diego; the nasopharyngeal (NP) region of COVID-19 positive patients (n=39, cohort 1) was swabbed by healthcare professionals and the swabs then stored in viral transport media (VTM) and transported to the lab on dry ice, according to CDC guidelines. In the second cohort (n=22, cohort 2), COVID-19 positive ICU patients (n=12) and healthcare workers (n=10) were sampled at the UC San Diego Medical Center in Hillcrest, San Diego, California; in lieu of performing uncomfortable nasopharyngeal sampling, the SYN swabs were used to collect nares samples by rotating the dry swab head in the nares for approximately 10 seconds and then immediately placed in 95% ethanol (EtOH), and transported to the lab on dry ice [5]. All collections were performed in accordance with approval of the UC San Diego Institutional Review Board under protocols #150275 and #200613.

Eluent nucleic acid extractions from both cohorts were performed on 200 μL of the swab eluent (either VTM or EtOH) using the Omega Mag-Bind^®^ Viral DNA/RNA 96 Kit (catalog# M6246-03), which only uses chemical lysis and does not include a bead beating step. For nucleic acid extraction from the swab head, the MagMAX Microbiome Ultra kit (Cat#A42357, Thermo Fisher Scientific) was used. Note, both kits are approved by CDC for SARS-CoV-2 diagnosis. To test the influence of storage media or extraction type (eluent vs swab head), we compared the extraction efficiency using the human RNase P (Rp) gene which is the internal control for all SARS-CoV-2 RT-qPCR tests as a proxy for biomass. All human specimens regardless of SARS-CoV-2 diagnosis will test positive for the Rp gene if they contain sufficient non-degraded nucleic acid. We compared the total concentration of Rp gene copies per extraction across the three groups using an ANOVA with Tukey post hoc test. Detailed descriptions of the sampling design can be found in Supplemental Table 1a.

For the direct comparison of SARS-CoV-2 extraction efficiency, we extracted eluent and swab head samples from a subset of COVID-19 positive patients (n=7) where the nares swab was stored in 95% EtOH. The SARS-CoV-2 RNA copies were quantified using RT-qPCR using the N1 primer. Comparisons of each pairwise sample was performed using a one-tailed paired Student’s *t*-test.. The experimental design and metadata from this first experiment is included as a Supplemental Table 1b. A follow up experiment was conducted to determine if alcohol based storage solutions degrade RNA or inhibit RNA extraction efficiency. To do this, we added the same about of human RNA to water (n=4), 95% EtOH (n=3), and 91% isopropanol (n=3). We then performed RNA extraction using the MagMAX kit followed by RT-qPCR to quantify the total amount of human RNA using the Rp gene. We calculate efficiency based on the quantified amount of starting RNA and compare these yields using ANOVA. Detailed descriptions of the sampling design can be found in Supplemental Table 1c.

### RT-qPCR for VTM and 95% EtOH comparison using synthetic-tipped plastic swabs

SARS-CoV-2 detection was performed following a miniaturized version of the CDC protocol. Each RT-qPCR reaction contained 4 μL RNA template, 100 nM forward and reverse primers, 200 nM probe, 3 μl TaqPath (catalog# A15299, Thermo), and RNase-free water to a total reaction volume of 10 μl. All primers and probes were ordered from IDT (catalog# 10006606). RT-qPCR was performed on the Bio-Rad CFX384 Touch Real-Time PCR Detection System following the CDC thermocycling guidelines. Serial dilutions of the Hs_RPP30 Positive Control plasmid (catalog# 10006626, IDT) or 2019-nCoV_N_Positive Control plasmid (catalog# 10006625, IDT) were included to extrapolate Rp gene and SARS-CoV-2 copy numbers, respectively. The SARS-CoV-2 N1 marker gene was used for detection and quantitation [12] (CDC 2019-Novel Coronavirus (2019-nCoV) Real-Time RT-PCR Diagnostic Panel. 2020 v134922).

### Evaluation of alcohol-based storage solutions for SARS-CoV-2 detection

The next experiment sought to determine if alcohol based storage solutions provide any protection against RNases. Since RNases are likely to be present in clinical samples such as nasopharyngeal or nares, we modeled this by spiking in RNaseA to determine if alcohols inhibit the RNases. To do this, we added human RNA (600 ng) along with an equal volume (5 μL) of SARS-CoV-2 RNA to nine tubes containing 95% EtOH and nine tubes containing 91% isopropanol. For each of the two alcohol groups we added either 2.5 (n=3) or 25 μg RNaseA (n=3) along with a positive control of no RNase added to assess any inhibition offered against RNase contaminants. The amount of RNaseA added is relatively high, as the lower amount is what is normally recommended for doing RNA removal during nucleic acid extractions. The experimental design and metadata from this first experiment is included as a Supplemental Table 1d.

### Validation of use of alternative swabs (testing inhibition of SARS-CoV-2 detection)

**Figure 1:**
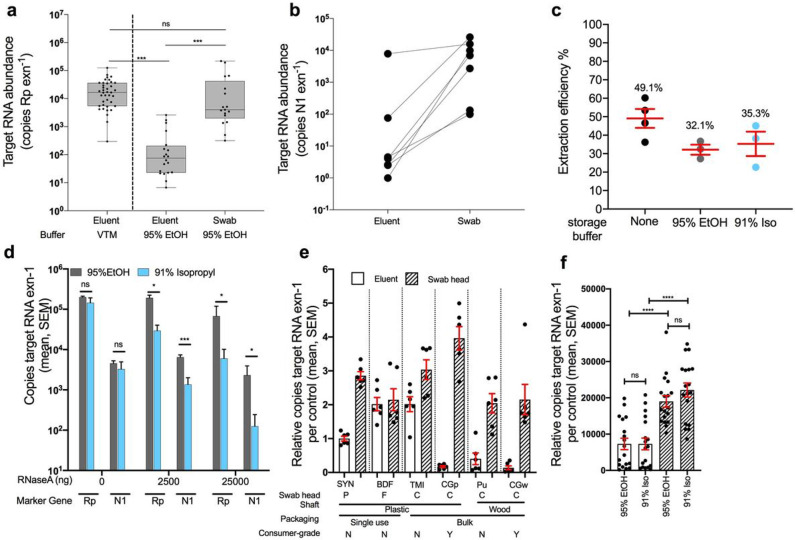
Validation of alternative swabs and storage buffer (95% EtOH and 91% isopropanol) in RNA recovery and detection of COVID-19. a) Human RNAse P gene (Rp) amplification was used to compare nucleic acid extraction efficiency across sample processing methods. Clinical gold-standard synthetic-tipped plastic-shaft NP swabs stored in VTM and extracted from 200 μL of eluent (left, n=39) have significantly higher copy numbers compared to 200 μL EtOH eluent from SYN nares swabs (middle, n=22), but not when extracted from the EtOH-preserved swab head (right, n=18). One-way ANOVA with Tukey’s multiple comparison VTM eluent vs EtOH eluent p=<0.001, EtOH eluent vs EtOH swab p<0.001, VTM vs EtOH swab p = 0.266. b) Extrapolated viral RNA copy number from COVID-19 positive nares samples collected with BD synthetic swabs in the hospital stored in 95% EtOH and extracted from either the eluent or swab from the same sample (n=24, one-tailed paired Student’s T-test p=0.032). c) Proportion of RNA recovered across three storage buffers: None, 95% EtOH, and 91% isopropanol using commercial human RNA added to storage buffers (ns, one-way ANOVA p>0.05). d) Evaluation of RNaseA inhibition by 95% EtOH (grey) and 91% isopropanol (blue) using either the human Rp or SARS-CoV-2 N1 primer set on control RNA added to each solution (unpaired t-tests of 95% EtOH vs 91% Iso per each marker at 0, 2500, 25000 ng RNaseA). e) Comparison human RNA recovery across six swab types (SYN=synthetic rayon ‘commercial’, BDF=BD foam ‘commercial’, TMI=BD TMI ‘commercial’, CGp=plastic ‘consumer-grade’, Pu=Puritan ‘commercial’, CGw=wood ‘consumer-grade’), extracted from 200μL eluent (blank bar) or the swab head. Recovery for each swab type is normalized to the CDC recommended method (eluent from PE swab). A ‘2’ would indicate there was 2x more RNA recovered whereas a 0.5 would indicate a 50% reduction in RNA recovery. f) Total RNA copies per extraction for all samples which are grouped by sample-type (eluent or swab head) and storage buffer (95% EtOH or 91% isopropanol). Pairwise comparisons performed within sample-type (not significant) and across sample-type controlling for storage buffer (Mann-Whitney, U=test statistic).

We tested a total of six swabs: CDC-compliant SYN swabs and five alternative swabs that included both plastic and wood materials for the shaft and synthetic, foam, or cotton materials for the swab head. The exact alternative devices used were ; sterile foam-head, plastic-shaft )(BDF) swabs (Flock PurFlock REF-25-3606-U-BT, Becton, Dickinson and Company); non-sterile cotton-head, plastic-shaft (TMI) swabs in use by The Microsetta Initiative (SKU#839-PPCS, Puritan Medical Products); non-sterile cotton-head plastic-shaft consumer-grade (CGp) swabs (Part #165902, CVS Caremark Corp.); non-sterile cotton-head wooden-shaft consumer-grade (CGw) swabs( Part#858948, CVS Caremark Corp.); and non-sterile cotton-head, wooden-shaft (Pu) swabs (REF-806-WC, Puritan Medical Products). The goal was to evaluate if RNA recovery is influenced by swab type or by extraction material (swab head vs. eluent). The standard protocol uses the eluent while extraction from swab head directly would be a new method. A total of six swab types were compared and all swabs were processed following the standard SARS-CoV-2 RT-qPCR protocol provided by the CDC [6]. To evaluate if the raw swab materials had any background contaminants such as RNase, which would decrease the sensitivity, we added 600 ng of purified, DNA-free human lung RNA (Cat#AM7968, Thermo Fisher Scientific) onto each of the six swab types in triplicate and immediately stored the swabs in two storage solutions (500 μL 95% EtOH and 500 μL 91% isopropanol). The experimental design is further highlighted in the Supplemental Table 1e-f. Note, that the data from Supplemental Table 1e-f corresponds to both the Figure 1e and Figure 1f subpanels and respective statistical comparisons. Although not included in the Supplemental table for conciseness, we included various controls. This included two sets of six, 10-fold serial dilutions of human RNA, four negative (swab only), and four positive (swab + 600 ng spiked human RNA + 5 μL spiked SARS-CoV-2 RNA [~20,000 copies per μL]) controls.

### Limit of detection comparison of swabs using floor as substrate

To estimate the limit of detection (LoD) and compare the viral yield across three swab types (SYN, CGp, and TMI), a serial dilution of viral particles was spiked onto floor swabs. In brief, separate 25 cm x 25 cm areas of the floor from a low-traffic common room inside the Marine Biology Research building at UC San Diego, a building with no SARS-CoV-2 research activities, were swabbed with a total of 24 swabs per swab type. Swabs were processed in groups of six by swabbing a quarter of that 625-cm^2^ space, with each swab ultimately covering an *ca.* 26-cm^2^ area, the similar surface area (25 cm^2^) used for detection of low biomass samples in JPL spacecraft assembly clean rooms based on previous work in the JPL spacecraft assembly facility [3]. Swabs were then stored at room temperature for *ca.* 1 hr in a 2-mL deep-well 96-well plate during transport back to a BSL-2 laboratory at UC San Diego. A single serial dilution of SARS-CoV-2 viral particles [BEI Resources: Cat# 52286, Lot# 70033548] was made at the following concentrations: 232000, 2320, 1160, 580, 290, 145, and 72.5 viral particles per μL. A total of 5 μL of each dilution, or water as a negative control, was pipetted onto each swab type in triplicate and then immediately placed into 95% EtOH. Swabs in EtOH were then stored overnight at −80°C until processing. Upon processing, an additional 24 ‘no swab’ controls were included whereby 5 μL of the dilutions were dispensed directly into the extraction plate lysis buffer. Samples were processed using the same nucleic extraction method as described for swab heads above, and eluted in 75 μL of elution buffer. For RT-qPCR, 5 μL of template was used for each marker N1 and Rp. To address potential issues of non-normality, total copies were compared across swab types at each individual dilution using Kruskal-Wallis tests with Benjamini-Hochberg FDR 0.05 post-hoc test.

### Patient and hospital environmental sampling

All study patients were hospitalized with clinical concerns for COVID-19 and received standard diagnostic testing. Study samples were collected from subjects' nares or hospital surfaces using three dry swab types (SYN, TMI, CGp) under the UC San Diego Institutional Review Board protocol #150275 and #200613. Both nasal samples and hospital surfaces were collected using three dry swab types (SYN, TMI, CGp). Nasal samples were collected by inserting the swab into one nostril to the depth of approximately 2-3 cm and rotated for 5-10 seconds. Hospital surfaces sampled included the floor inside the patient’s room (*ca*. 625-cm^2^ area) and the patient’s bedrail. All swabs were immediately placed in a collection tube containing 0.5-1.0 mL 95% EtOH, stored on dry ice, and processed for RNA or total nucleic acid extraction.

### Extraction and RT-qPCR of hospital swabs and controls

All swab comparison- and hospital samples were processed according to the manufacturer’s protocol using the MagMAX Microbiome Ultra kit (Cat#A42357, Thermo Fisher Scientific), and eluted into 70 μL buffer. For RT-qPCR, 5 μL sample was processed using the standard SARS-CoV-2 protocol provided by the CDC (Cat# 2019-nCoVEUA-01,[13]).

### Microbiome processing and analysis

A subset of 40 samples were processed for 16S rRNA gene sequencing using established EMP protocols [14]. These included 18 floor samples, 21 nasal samples, and 1 negative control. Floor samples included all triplicates from the three swab types (SYN, TMI, and CGp) from two patient rooms (patient 7 and 18). The nasal samples included triplicates of all three swab types from patient 1, triplicates of SYN and CGp from patient 7, and triplicates of SYN and TMI from patient 18. The same previously extracted nucleic acid template, which was concurrently used for RT-qPCR, was used as template for 16S rDNA sequencing library generation (amplifying the DNA). Specifically, 0.4 μL of nucleic acid was processed in 10 μL 16S rRNA PCR reactions following the miniaturized protocol [15] using the 515f/806r EMP primers, and sequenced on an Illumina MiSeq {Citation}[16-19]. Samples were then processed in Qiita (Study ID 13275) [20] and analyzed using the QIIME2 2020.6 [21,22] pipeline with Deblur [23] 1.1.0 as the method of sub operational taxonomic unit (sOTU) generation. Samples were visualized in PCoA plots in Qiita using EMPeror [24]. Beta diversity was calculated using unweighted Unifrac and compared with PERMANOVA (999 permutations).

### Statistics and visualizations

Visualizations and statistical comparisons performed using PRISM 8.0 and the limit of detection determination were consistent with CDC recommendations whereby samples with a Ct value greater than 40 were omitted (Cat# 2019-nCoVEUA-01) [13].

## Results

Our experimental design sought to answer three primary questions: whether the efficacy of SARS-CoV-2 detection is influenced by the following three variables: 1) does the swab storage solution (95% EtOH vs 91% isopropanol) impact the sensitivity of detection; 2) which sample fraction, swab head or eluent, provides better detection fidelity; and 3) does the swab head material type matter? To do this we designed a series of experiments to compare RNA recovery as measured by RT-qPCR using multiple swab types and storage solutions. We additionally performed environmental sampling in a hospital environment with a subset of swab types for comparison.

### Feasibility of 95% EtOH for sample storage and extraction from use of swab head rather than eluent

To evaluate the feasibility of switching from VTM to a more readily-available, viral-inactivating sample collection solution, we compared the extraction efficiency of synthetic-tipped plastic-shafted nasopharyngeal (NP) SYN swab samples stored in VTM versus nasal samples collected using SYN swabs stored in 95% ethanol (EtOH) collected from two separate cohorts (Methods). When mirroring the CDC protocol, which calls for extraction from 200 μL of the eluent from VTM surrounding NP swabs, we had significantly lower recovery of human RNA in 95% EtOH eluent compared to the RNA copy concentrations in the eluent of SYN swabs collected in VTM from a separate cohort of COVID-19 positive patients (Figure 1a; one-way ANOVA with Tukey’s multiple comparison, VTM eluent vs. EtOH eluent p<0.001). However, similar levels of human RNA were recovered when extracting from the EtOH-preserved swab head itself (Figure 1a; one-way ANOVA with Tukey’s multiple comparison, VTM vs. EtOH swab p=0.3). In a subset of seven COVID-19 patient nares samples stored in 95% EtOH, we also detected significantly higher SARS-CoV-2 viral load in RNA extracted from the swab head versus eluent (Figure 1b; one-tailed paired Student’s *t*-test p=0.03).

To more quantitatively determine the effects of alcohol-based preservation media, we extracted RNA from a pure, commercial sample of human RNA added to water, EtOH, or 91% isopropanol, and found no impact on extraction efficiency (Figure 1c; one-way ANOVA, p>0.05). Next, we examined whether alcohol storage solutions had any protective properties of RNA, specifically a possible inhibitory effect on RNases that might be present in the environment. If alcohol inhibits the RNaseA, one would expect to see similar amounts of RNA as without RNaseA added in control experiments. In the presence of abundant RNaseA added to the solution, 95% EtOH protected both human RNA and SARS-CoV-2 RNA better than 91% isopropanol. Only a moderate decrease in total RNA recovery was observed, at the most extreme concentration of 25 μg per reaction, which is equivalent to the standard amount used for RNA removal during DNA extraction (Figure 1d).

### Comparison of alternative swab types against standard CDC approved synthetic swab

Given that the performance of eluent vs. swab-based extractions in each alcohol may depend on the swab tip and body composition, we next tested RNA recovery from both the swab head and the surrounding eluent from a range of medical- and consumer-grade swabs (Methods) (Figure 1e). The RNA yield was highest from swab heads compared to eluent regardless of the swab type and whether stored in 95% EtOH (p<0.0001, U=37, Mann-Whitney) or 91% isopropanol (P<0.0001, U=28, Mann-Whitney) (Figure 1e-f). The storage solution did not impact RNA quality (Supplemental Figure 1b, Mann-Whitney, p>0.05), although swab type had a minor impact (Supplemental Figure 1c, Kruskal-Wallis p=0.03, KW=12.17) [25]. To compare impacts of various alternative swabs, we normalized the recovery of each test to SYN eluent, indicated by ‘1’ (Figure 1e), which is the standard CDC approved method. Thus, any sample with a value greater than 1 would indicate an enhanced recovery of RNA, whereby less than 1 indicates a lower recovery of RNA compared to the standard. The RNA recovery ratio of swab-to-eluent and total yield varied among swab type (p<0.0001, KW=28.37, Kruskal-Wallis for eluent, and p<0.0001, KW=15.43, Kruskal-Wallis for swab heads) (Supplemental Figure 2). This difference in performance may relate to the differences in observed adsorption capacity across swab types (Shapiro-Wilkes p=0.1, w=0.8357; ANOVA p=0.0001, F=7.5, R^2^=0.56). TMI adsorbed the least (84.5 μL, 20.4; mean, SD) followed by plastic shafts (SYN: 141 μL, 23.1; CGp: 143.3 μL, 29.9) (Supplemental Figure 3). CGp swabs had the highest recovery of RNA from the swab head, while TMI swabs had the highest overall recovery of RNA when combining both eluent and direct swab extractions (Figure 1e, Supplemental Figure 2).

### SARS-CoV-2 limit of detection comparison across swab types

**Figure 2:**
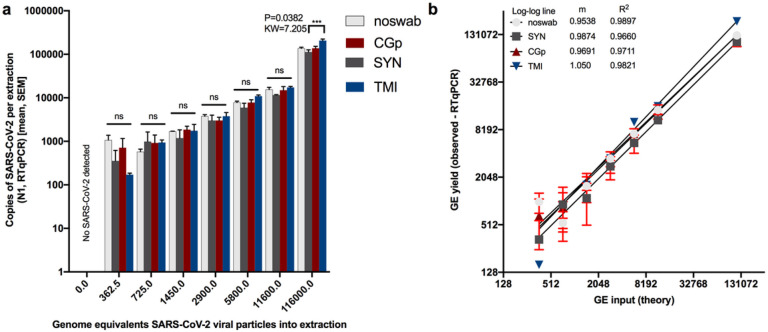
Limit of detection of SARs-CoV-2 viral particles across swab types (Synthetic rayon ‘SYN’, CGp, and TMI). “Noswab” refers to direct extraction of viral particles. a) Comparisons of total RNA recovery per extraction across swab types including ‘noswab’ performed at each dilution (Kruskal Wallis test). Comparison of (theory input Genome Equivalents ‘GE’) to measured GE of triplicates (mean, SEM) by RTqPCR of SARs-CoV-2. Non-linear regression analysis of each dilution series for noswab, SYN, CGp, and TMI swabs.

We next assessed whether the swab type used would impact the recovery of SARS-CoV-2 and alter the limit of detection when using non-CDC-recommended swabs (CGp or TMI swabs compared to SYN swabs). All negative controls for floor swabs were indeed negative for SARS-CoV-2 using N1 and N2 (Supplemental Table 2, Figure 2) and all ‘no-swab’ controls which only had SARS-CoV-2, were negative for human Rp (Supplemental Table 2). For the ‘no-swab’ and TMI swab, SARS-CoV-2 was detected in all of the three replicates at the lowest input of 362.5 genome equivalents ‘GE’, whereas the lowest dilution for all three replicates to be positive for CGp and SYN swabs was 1450 GE (Supplemental Table 2, Figure 2a). This suggests the limit of detection for neat and TMI swabs is likely between 0 and 362.5 GE per reaction whereas both CGp and SYN swabs were less sensitive with an expected limit between 750 and 1450 GE per reaction. There was a strong correlation between the input or theoretical GE and the measured GE with slopes all greater than 0.95 and the R^2^>0.96. Despite TMI swabs appearing to have the best overall performance in SARSs-CoV-2 detection followed by SYN swabs and then CGp swabs, the total viral yield did not differ across swab types at the lowest dilution of 362.5 (P>0.05, Kruskal-Wallis test) (Figure 2a). Specifically, multiple post-hoc comparisons showed that variation across swab-type only existed at the highest concentration (116,000 GE) with the TMI swabs having a higher viral recovery compared to SYN swabs (P=0.04, KW=7.21) (Figure 2). Rp yield was also compared across swab types and across viral inputs to characterize the variation in input biomass. For each swab type, human RNase P (Rp) gene was equally detected across the titrations indicating the swab method was sufficiently controlled (Supplemental Figure 4a). Swab type, however, did suggest that the Rp gene was highest in the SYN swab as compared to the CGp and TMI swabs (Kruskal-Wallis: P<0.0001, KW=41.41) (Supplemental Figure 4b). This result suggests that SYN swabs may adsorb more biomass. However, when we compared the variation in Cq values of hospital samples of nares and floor from the same hospital using SYN swabs, we observed Rp gene values that varied over six orders of magnitude (Supplemental Figure S5), much greater than the three orders of magnitude observed across swab types. Specifically, for floor samples, the Rp gene yield (copies per extraction) range across swab types was 149-3368 copies for SYN swabs, 0-3980 for CGp swabs, and 0-207 for TMI swabs.

### Hospital proof of concept study

**Figure 3:**
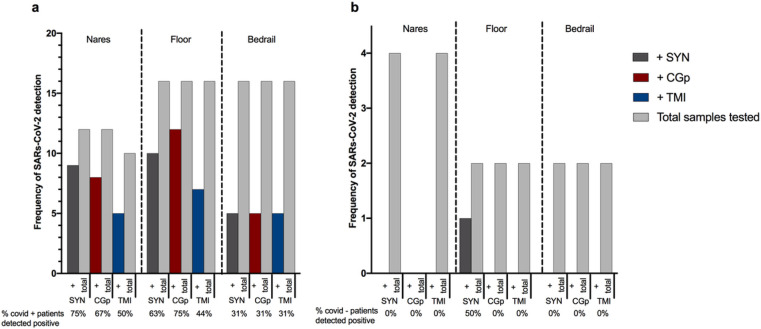
Comparison of CDC approved SYN swabs, consumer-grade CGp and bulk TMI swab congruence compared to clinical-grade hospital tests using synthetic-tipped plastic shafted NP swabs for twenty participants in the clinical setting. a) SARs-CoV-2 positive patients (n=16) sampled with three swab types across three environments: nares, floor, and bedrail. ‘+’ samples (dark grey = SYN, red = CGp, blue = TMI) refer to samples which tested positive for SARs-CoV-2 out of the total samples tested for that particular swab type (light grey bar). Percentage of positive tests per swab type are below x axis for each environmental sample. b) SARs-CoV-2 negative patients (n=4) with three swab types across three environments: nares, floor, and bedrail. Same nomenclature as above.

Based on the results from these initial experiments, we conducted a proof-of-concept study in the clinical setting by performing RT-qPCR for the SARS-CoV-2 N1 amplicon and Rp gene on RNA extracted from the swab head of nasal samples collected using TMI and/or CGp swabs alongside the recommended SYN swabs. Of the 20 participants sampled, 16 tested positive for SARS-CoV-2 at admission and were designated as COVID-19(+). The average time from diagnosis to sampling was *ca.* 4.2 days, with a NP swab test occurring within 72 hours of the time of nasal sampling. Of the 12 nasal samples using the SYN swab preserved in EtOH from COVID-19(+) patients, nine were positive for the presence of SARS-CoV-2 or a false negative rate of 25% (Figure 3a) compared to 14/16 SARS-CoV-2 positive NP swabs for the same group of patients, a false negative rate of 12.5%. For CGp and TMI swabs, 8/12 and 5/10 were positive for nares, respectively (Figure 3a). These rates of false negatives are similar, as compared to the 37.5% false negative rate reported for plastic-shafted synthetic-tipped nasal swabs collected in VTM and extracted from the eluent. As the degree of viral shedding is known to vary over the course of the disease [26], we compared the performance in the subset of COVID-19(+) patients with an NP-positive swab result within 72 hours of the time of sampling, and observed reduced false negative rates of 18.2% (SYN), 25% (TMI), and 30% (CGp). We next compared success rates across swab samples from the built environment. On the floor samples, the CGp swabs had the highest success rate at 75% in detection of SARSs-CoV-2 from SARSs-CoV-2 positive patient rooms whereas SYN swabs detected SARSs-CoV-2 in 63% of rooms, and TMI in 44% of rooms (Figure 3a). Bedrail samples had the lowest frequency of detection, 5/16 (31%), for each swab type (Figure 3a). For SARSs-CoV-2 negative patients admitted to the same hospital for other reasons, all nares and bedrail samples were negative, whereas one floor sample using the SYN swab detected SARSs-CoV-2 (Figure 3b).

The observed differences in detection among nares and environmental samples, taken in context of our previous experimental results demonstrating that the swab type does not inherently impact SARS-CoV-2 detection, suggests that variation in sample collection from the nares and other environmental samples has an important role in detection sensitivity. When swabbing an environmental surface or body site (i.e., nares), there is inherent variation in the swabbing event which can be attributed both to stochastic differences in biomass (i.e. human cells, dust, etc.) present and collected as well as the downstream processes such as nucleic acid extraction and RT-qPCR. To evaluate if certain sampling locations or swab types were more variable than others, we calculated the intra-assay coefficient of variance (CV) of the Rp gene Cq values. When comparing the variation across sample types (environmental samples: bedrail and floor vs. nares swab head and nares eluent), the CV was significantly higher in floor (Mann Whitney p=0.0056), patient nares eluent (P=0.0017), and patient nares swab (Mann Whitney, P=0.0018) when compared to control (human RNA spike-in) samples. The median difference in nares swabs was greatest with a median difference in variance of 2.5 compared to controls (Supplemental S6a). In this case, the CV of the positive control samples would demonstrate the variance in combined extraction and RTqPCR thus is indicative of the total variance in molecular sample processing. Next, we stratified for swab types. Swab types also demonstrated an effect with the inter-swab variation between values measured with CGp and SYN along with CPg and TMI swabs for controls and patient samples being significant, respectively (Mann Whitney: P=0.0012, P=0.0127) (Supplemental Figures S6b). Overall these findings demonstrate how variability in a given sampling (swabbing) event can influence SARs-CoV-2 detection.

### Microbiome analysis

**Figure 4:**
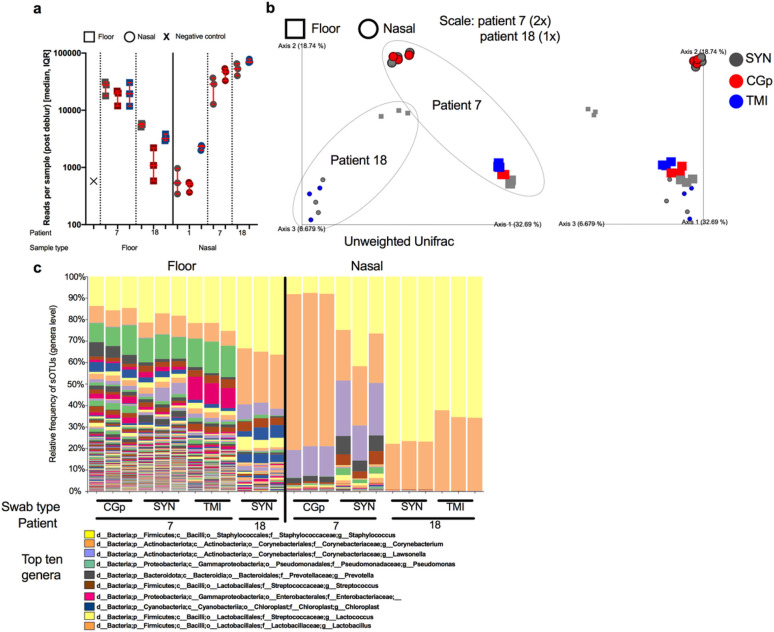
Microbiome 16S rRNA sequencing validation across sample types, patients, and swab types. a) Total number of reads per sample (40 samples sequenced) after processing through deblur pipeline stratified by sample type (floor ‘square’ vs nasal ‘circle’), patient number (1, 7, and 18) and colored by swab type (SYN = grey, CGp = red, TMI = blue). Error bars represent median, IQR for triplicate biological replicates per sample. b) Unweighted UniFrac PCoA plot of samples rarified to 5000 reads. Enlarged samples (2x) indicate patient 7 whereas (1x) indicates patient 18. Swab types are colored (SYN = grey, CGp = red, TMI = blue) and shapes (floor ‘square’ vs nasal ‘circle’) indicate sample type. Grey dotted line goes around each patient. c) Stacked bar plot collapsed at genera level with top ten abundant genera labeled in the legend.

To determine the feasibility of co-opting nucleic acid for microbiome processing, we processed a subset of samples (n=40) spanning a total of three patients, two sample types (floor and nasal) and the three swab types. After processing with Deblur, the total number of reads per sample were compared (Figure 4a). Read counts were highly variable across sample types and for each patient but were consistent within the swab types for each comparison. For floor samples in patient room 18, SYN swabs had the highest number of reads followed by TMI and CGp. For nasal samples however, patient 1 had the higher read counts from TMI while patient 7 and 18 both showed slightly lower read counts for SYN swabs as compared to alternative swabs. The differences were minor however and are primarily differentiated by patient room (Figure 4a). After rarifying to 5000 reads, a PCoA plot was generated from using unweighted UniFrac distances (Figure 4b). Samples which were collected using different swab types clustered together when controlling for patient room and sample type, suggesting that the swab type used does not have impact the microbiome analysis (Figure 4b). When analyzing all samples together, sample_type (floor vs nasal) and patient number (7 vs 18) were both significant drivers of the microbiome community (sample_type: PERMANOVA n=24, group=2, P=0.001, F=6.94; patient_num PERMANOVA n=24, group=2, P=0.001, F=6.92) whereas swab type did not have an effect (P=0.164). Distances between swab types were lower than distances between patients for both floor (Supplemental Figure S7a) and nasal (Supplemental Figure S7b) samples, with patient 7 exhibiting higher variation than patient 18. Floor samples generally had a higher microbial diversity compared to nasal swabs, with *Staphylococcus, Corynebacterium, Pseudomonas, Streptococcus,* and Enterobacteriaceae being the more dominant taxa. Nasal samples however were mostly enriched by either *Staphylococcus* or *Corynebacterium*, with patient 7 having a higher abundance of *Lawsonella* (Figure 4c).

## Discussion

When assessing whether it will be possible to adapt collection methodology to enable more affordable, more widely available, and more inter-assay compatible collection methods for SARS-CoV-2 monitoring, it is key to understand the feasibility of using both alternative swabs and sample storage solutions. Here we provide evidence that the variation observed in a given SARS-CoV-2 experiment is primarily driven by the time and method of sample collection rather than by the swab type, storage solution, and subsequent extraction and RT-qPCR. A critical caveat of our storage solution comparison was that different patient samples were used. An ideal experiment would be to collect two swabs from the same person and store each swab in two different buffers (VTM vs. 95% EtOH). However, the relative sizes of the nostril and swab heads would necessitate sequentially swabbing the same nostril or using a different swab in each nostril. The former approach would introduce the variable of reduced biomass from the first sampling, whilst the second would introduce the variable of nostril-nostril variation in biomass. Because our subsequent experiments showed equal biomass extraction efficiencies when compared to control extractions, we did not follow up on these additional experiments. In addition, we demonstrate that variability in the hospital sampling was primarily driven by the actual sample collection rather than swab type. When using alcohol-based storage solutions, we demonstrate that the nucleic acid or viral particles tend to become enriched on the swab head rather than the eluent and thus we recommend extracting directly from the swab head itself. We demonstrate that RNA can be successfully extracted from consumer-grade swabs stored in alcohol without compromising RNA integrity or yield. Of note, wooden-shafted swabs performed poorly only when extracting from the eluent, suggesting that RNA adsorption onto the shaft, rather than RT-qPCR inhibitors, may be the source of interference with current eluent-based testing methods for this swab type. As cotton-tipped swabs and alcohol-based storage solutions are compatible with standard microbiome- and metabolome analyses not feasible with VTM, these alternatives could enable more widespread assessment of the microbial context of SARS-CoV-2 RNA in human and environmental samples, including associated microbiome features.

We also provide preliminary evidence that nasal samples collected using more widely available, consumer-grade, cotton-tipped swabs can be used to detect SARS-CoV-2 in the clinical setting. As cotton-tipped TMI swabs had only a marginally reduced performance compared to CDC-compliant SYN swabs for nasal samples compared to NP results, these swabs have potential as an attractive alternative for methods such as metabolomics that are complicated by the background from incompatible with synthetic-tipped swabs, as well as suggesting that the pool of available collection consumables could be expanded beyond medical-grade materials. Notably, this variation is less than that observed when comparing different methods for assessing the presence of SARS-CoV-2. Larger-scale testing will be needed to expand and confirm these findings, but our data suggests that these two swab types, in either 95% EtOH or isopropanol, would provide a valuable starting point.

When considering environmental sampling, our data suggest that TMI and CGp swabs may outperform or at least are similar to, CDC-compliant SYN swabs for collecting samples to detect SARS-CoV-2 from floor samples. We provide molecular evidence demonstrating the feasibility of detecting SARS-CoV-2 from floor samples with a limit of detection (*ca.* 362.5 copies per extraction for TMI) and (750-1450 copies per extraction for CGp and SYN swabs) similar to that of other published studies (500 copies per extraction) [27]. Additional testing using pre-wetted swab heads, as performed in other built-environment studies [28-31], is warranted to determine if this would improve the ability of all swab types to detect SARS-CoV-2 in the hospital room environment. The detection of SARS-CoV-2 on *ca.* 50% of COVID-19(+) participants’ bedrails and *ca.* 75% of floors, as well as the detection of SARS-CoV-2 on the floor of one non-COVID patient, suggests a potential reservoir for infections. Further studies should be completed to quantify the infectivity of these viral particles which could lead to policy implications such as increased cleaning measures. Indeed, the floor may be a potentially important reservoir for viral exposure, as shoe-covers are not currently recommended by the CDC or WHO. However, additional testing is needed to determine whether viable virus particles remain on these surfaces. Since detection largely does not differ across swab types, this suggests that differences seen in the quantitation of SARS-CoV-2 in the clinic in a given floor or nasal sample is due to variation in the swabbing event itself rather than a molecular processing problem. Because of this, we recommend standardization in medical devices used to collect both nasal and environmental samples specific to SARSs-CoV-2 to improve overall accuracy. Lastly, our efforts to quantify the total noise in a given sampling event and sample processing itself demonstrate how variation in the act of swabbing combined with sample processing may lead to variance and at times lower than expected specificity.

Secondary infections are an important and significant contributing factor to morbidity and mortality in COVID-19 patients [32,33]. With metagenomics assays becoming more common for infectious disease diagnostics in the clinic [34-36], developing molecular methods which enable simultaneous viral detection and metagenomic analysis is critical for understanding disease progression in at-risk populations. Since the storage method is a critical step in preserving microbiome integrity with 95% ethanol as a stable solution [37], our results further demonstrate and open the door for multi-omics processing and analysis of SARS-CoV-2 samples.

## Conclusions

In summary, our results suggest detection of SARS-CoV-2 RNA in the environment could be performed using less expensive, consumer-grade materials and alcohol-based storage solutions. With the materials examined in this study, it is further conceivable that patients could collect samples from themselves, their environments at home, or their place of work, dramatically expanding the ability to deploy widespread methods for monitoring and predicting outbreak events. Additional confirmatory studies using consumer-grade swabs would greatly support COVID-19 screening worldwide, particularly in resource-limited communities.
